# 

**Published:** 1894-11-17

**Authors:** 


### CONTENTS.

**Albuminuria and Life Assurance** - - - - - - 109
**The Czar's Illness and Its Lessons** - - - - 110
**On Modern Progress in Ophthalmic Medicine and Surgery.** By R. Brudenell Carter, F.R.C.S. Iritis (*contd.*) - 111
**The First Century of English Medical Literature.—X.** Theories of Sanitation - - - - - - - - 112
**Annotations.**—Text Book Surgery—"Cures" for Drunkeness—Glasgow's Hospital Sunday - - - - - - - 113
**Notes and News** - - - - - - - - - - 114
**Editor's Letter-Box** - - - - - - - - - 124
**Scraps and Gleanings** - - - - - - - - - 124
**Medical Progress and Hospital Clinics:**
Chronic Catarrh of the Middle Ear.—V. (*continued*) - - - 115
Progress in Medicine.—Infective Diseases (*concluded*) - 117
Progress in Surgery.—Surgery of the Intestines (*continued*) - 119
**The Institutional Workshop:**
Within the Hospitals - - - - - - - - - 121
Canadian Hospitals - - - - - - - - - 122
Practical Departments.—Electric Lighting for Hospitals (*continued*) - - - - - - - - - 122
Around the Hospitals - - - - - - - - - 123
**The Book World of Medicine and Science** - - - - 125

### EXTRA SUPPLEMENT.—THE NURSING MIRROR.

**News from the Nursing World.**—Students or Nurses?—Our Christmas Competition—Only Human Lives!—Rushden—Good Work Appreciated—District Nursing at Rugby—The Fifth Time of Asking—Activity at Belfast—Provision for Sickness—Good Times—Outwardly Clean—Nurses at Swansea—Short Items - - - - - - - - - - xli

**Our American Letter** - - - - - - - - - xliii

**French Schools for Trained Nurses: Their Origin and Organisation** - - - - - - - - - xliii

**Nursing in Ireland** - - - - - - - - - xliv

**The American Woman at Home.** I.—Her Home - - xlv

**Appointments** - - - - - - - - - - - xliv

**Notes and Queries** - - - - - - - - - xliv

**Dress and Uniforms—Where to Go** - - - - - xlvi

